# Supplementary material for: Long-Term Breast Cancer Outcomes of Pregnancy-Associated Breast Cancer (PABC) in a Prospective Cohort
Source: Cancers (Basel). 2022 Oct 4;14(19):4839. doi: 10.3390/cancers14194839 (PMC9564289; doi:10.3390/cancers14194839)
Supplement: Supplementary file 1 [file cancers-14-04839-s001.zip › Supplementary file.pdf]

Supplementary Table S1. Receptor status and treatments in the metastatic setting (n = 42)

Characteristics	PABC group (n = 11)	Non-PABC group (n = 31)	P value
Receptor status			
HR+/HER2-	4 (36.3)	15 (48.4)	0.665*
HR+/HER2+	3 (27.3)	5 (16.1)	
HR-/HER2+	0	3 (9.7)	
Triple negative	4 (36.4)	8 (25.8)	
Metastatic sites number	3 (2-4)	3 (1-4)	0.527
Progression during neoadjuvant chemotherapy	2 (18.2)	8 (25.8)	0.999*
Breast surgery in metastatic setting	1 (9.1)	2 (6.5)	0.999*
First line initial response to systemic therapy			
Complete response	1 (9.1)	0	0.396*
Partial response	2 (18.2)	7 (22.6)	
Stable disease	0	1 (3.2)	
Progressive disease	8 (72.7)	23 (74.2)	

Data are shown as n (%) per each group or median (range).

* P value was calculated from the Fisher's exact test.

PABC, pregnancy-associated breast cancer; HR, hormone receptor; HER2, human epidermal growth factor receptor 2.

Supplementary Table S2. Subgroup analysis of the PABC group (*n* = 93)

Characteristics	Diagnosed during pregnancy (*n* = 28)	Diagnosed within 1-year postpartum (*n* = 65)	*P* value
Age at diagnosis (years)	33 (28-43)	35 (26-43)	0.523
Gestational weeks at diagnosis	20 (4-38)	-	
First trimester	9 (32.1)	-	
Second trimester	12 (42.9)	-	
Third trimester	7 (25.0)	-	
Gestational weeks at delivery[a]	37 (30-40)	-	
Termination of pregnancy	5 (17.9)	-	
Postpartum months at diagnosis	-	6 (0.25-12)	
Receptor status			
HR+/HER2-	14 (50.0)	28 (43.1)	0.261*
HR+/HER2+	8 (28.6)	10 (15.4)	
HR-/HER2+	1 (3.6)	6 (9.2)	
Triple negative	5 (17.9)	21 (32.3)	
Treatment setting			
Neoadjuvant	13 (46.4)	36 (55.4)	0.685
Adjuvant	11 (39.3)	22 (33.8)	
Palliative	4 (10.7)	7 (10.8)	
Neoadjuvant chemotherapy			
AC	7 (53.8)	-	
AC + docetaxel	6 (46.2)	20 (55.6)	
AC + docetaxel + platinum	-	1 (2.8)	
AC + docetaxel + trastuzumab	-	8 (22.2)	
TCHP	-	4 (11.1)	
Others	-	3 (8.3)	
pCR	2 (7.1)	12 (18.5)	0.215
Adjuvant chemotherapy	15 (53.6)	26 (40.0)	0.260
Adjuvant trastuzumab	6 (21.4)	14 (21.5)	0.999
Adjuvant endocrine therapy	17 (60.7)	38 (58.5)	0.650
Adjuvant radiotherapy	18 (64.3)	42 (64.6)	0.816

Data are shown as n (%) per each group or median (range).

* *P* value was calculated from the Fisher's exact test.

[a] Data are from 23 patients excluding 5 patients who terminated pregnancies.

PABC, pregnancy-associated breast cancer; HR, hormone receptor; HER2, human epidermal growth factor receptor 2; AC, anthracycline and cyclophosphamide; TCHP, docetaxel, carboplatin, trastuzumab and pertuzumab; pCR, pathological complete response.

Supplementary Figure S1. Study design.

YBC, young breast cancer; PABC, pregnancy-associated breast cancer.

Supplementary Figure S2. Kaplan-Meier curves of survival of the PABC group. (A) Disease-free survival. (B) Overall survival.

PABC, pregnancy-associated breast cancer.
