# Supplementary figures and images for: Long-Term Breast Cancer Outcomes of Pregnancy-Associated Breast Cancer (PABC) in a Prospective Cohort
Source: Cancers (Basel). 2022 Oct 4;14(19):4839. doi: 10.3390/cancers14194839 (PMC9564289; doi:10.3390/cancers14194839)

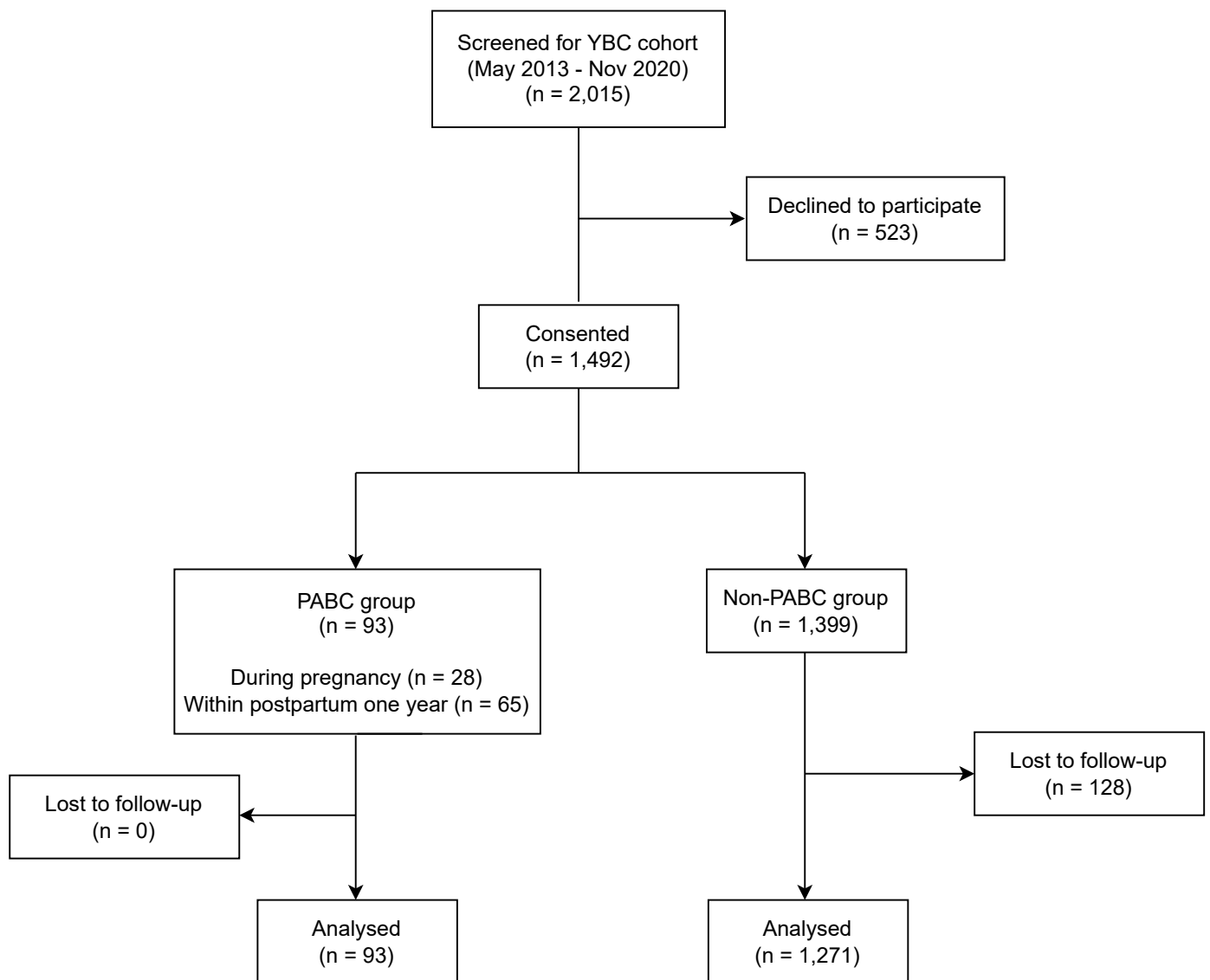

Supplement: Supplementary file 1 [file cancers-14-04839-s001.zip › Supplementary Figure S1.pdf]

A

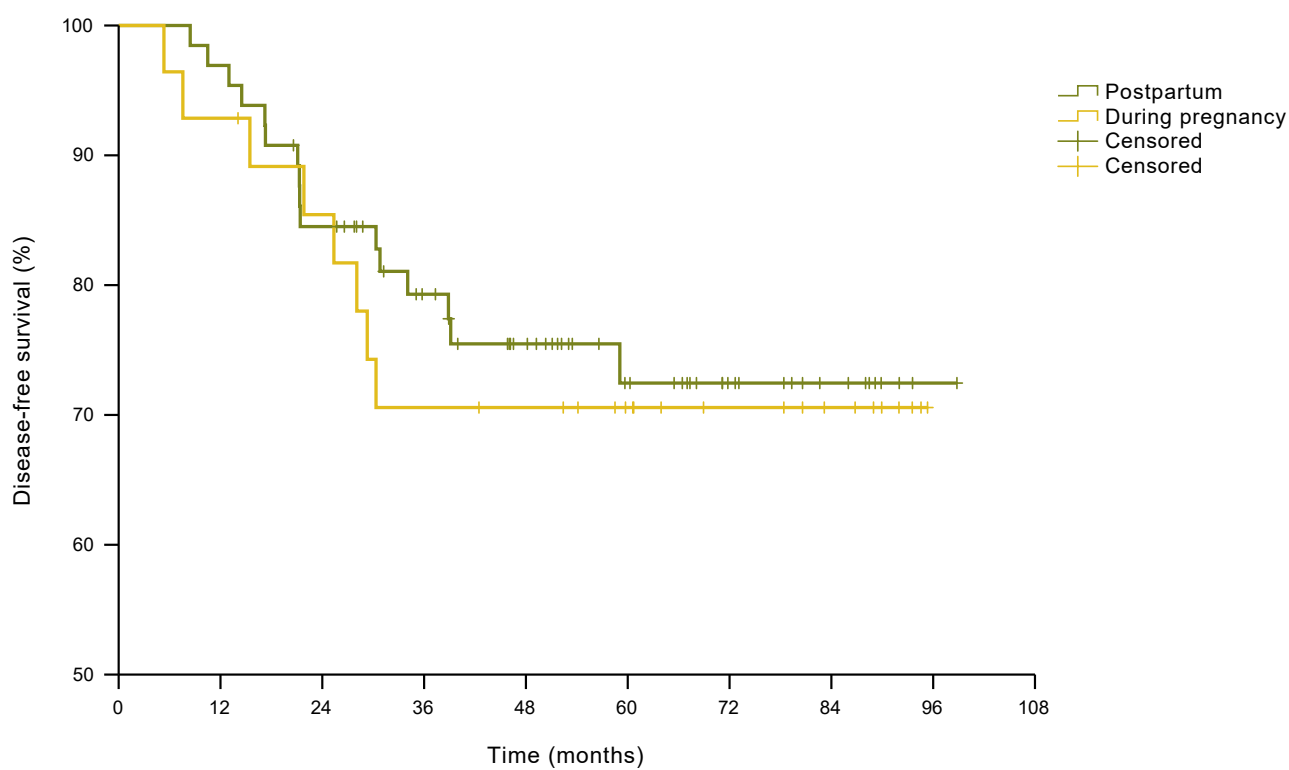

|                  |    |    |    |    |    |    |    |   |   |   |
|------------------|----|----|----|----|----|----|----|---|---|---|
| No. at risk      |    |    |    |    |    |    |    |   |   |   |
| Postpartum       | 65 | 63 | 54 | 43 | 34 | 23 | 14 | 8 | 1 | 0 |
| During pregnancy | 28 | 26 | 23 | 19 | 18 | 14 | 10 | 7 | 0 | 0 |

B

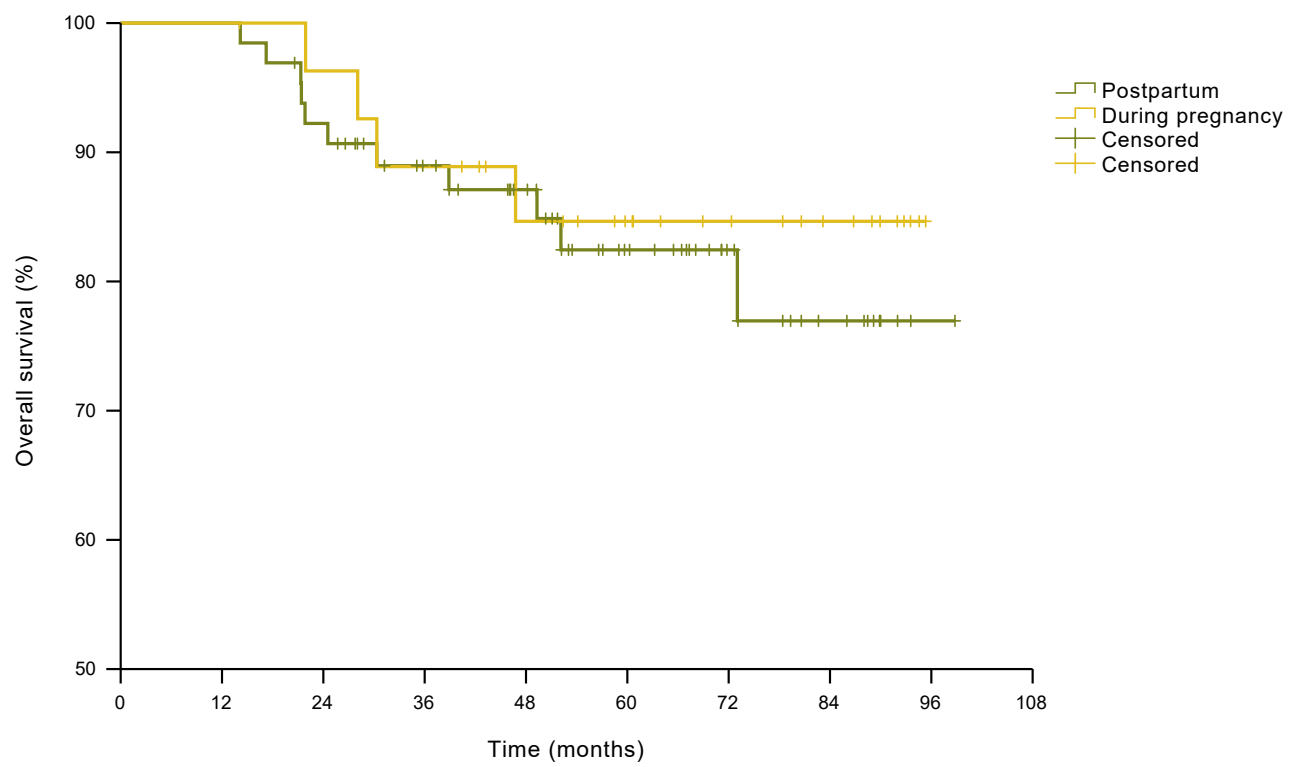

|                  |    |    |    |    |    |    |    |   |   |   |
|------------------|----|----|----|----|----|----|----|---|---|---|
| No. at risk      |    |    |    |    |    |    |    |   |   |   |
| Postpartum       | 65 | 65 | 59 | 49 | 41 | 27 | 16 | 9 | 1 | 0 |
| During pregnancy | 28 | 28 | 26 | 24 | 20 | 16 | 12 | 8 | 0 | 0 |

Supplement: Supplementary file 1 [file cancers-14-04839-s001.zip › Supplementary Figure S2.pdf]
